# Cultivation to consumption: strengthening bacterial safety in plant-based nutraceuticals

**DOI:** 10.3389/fmicb.2025.1698580

**Published:** 2025-12-18

**Authors:** Ashish Gaur, Nishant Singhal, Harsh Vardhan, Rajul Jain, Yograj Bist, Naresh Kumar Wagri

**Affiliations:** 1 Department of Biosciences, Graphic Era (Deemed to be) University, Dehradun, India; 2 Department of Biochemistry, Banaras Hindu University, Varanasi, India; 3 School of Life Sciences (SLS), Central University of Gujarat, Gandhinagar, India; 4 Department of Zoology, Dayalbagh Educational Institute (DEI), Agra, India; 5 Department of Biotechnology, Graphic Era (Deemed to be) University, Dehradun, India; 6 Department of Materials Science and Engineering, School of Industrial Engineering and Management, KTH Royal Institute of Technology Stockholm, Sweden

**Keywords:** cultivation, harvesting, microbial contamination, nutrition, plant-based nutraceuticals

## Abstract

Plant-based nutraceuticals are increasingly recognized for their bioactive compounds that promote health and assist in preventing chronic diseases. However, the rising demand has raised concerns about microbial safety, as contamination can occur at multiple stages of the production process-ranging from cultivation and harvesting to processing, storage, and distribution. Pathogens such as *Escherichia coli, Salmonella, Listeria monocytogenes*, and toxin-producing fungi pose risks to product quality, threaten consumer health, and contribute to antimicrobial resistance. This review provides a comprehensive overview of the sources and types of microbial contamination, associated health risks, and the shortcomings of conventional control methods. It highlights recent advancements in safety techniques, including cold plasma, ultraviolet light treatment, high hydrostatic pressure, nanocoatings, probiotic biocontrol, and AI-driven microbial monitoring. Additionally, the analysis addresses the role of regulatory frameworks, quality assurance practices, and consumer education as integral elements of a unified safety approach. It integrates technological progress, regulatory perspectives, and consumer behavior to offer a detailed guide for ensuring the microbial safety of plant-derived nutraceuticals, thereby fostering confidence in these products from production through to consumption.

## Graphical abstract

**Figure:**
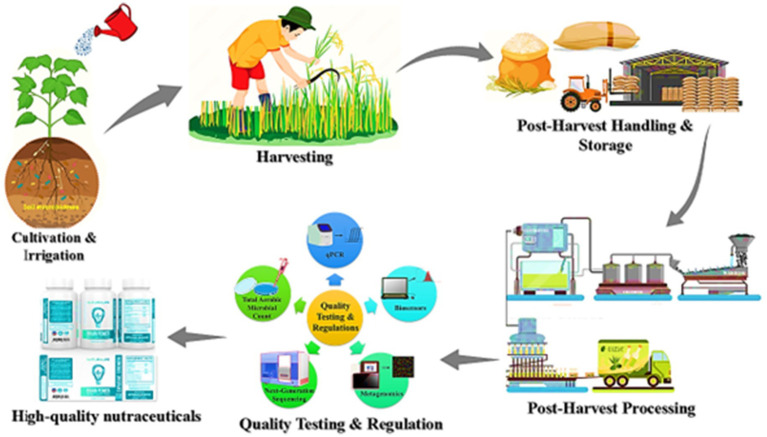

## 1 Introduction

Plant-based nutraceuticals have garnered significant attention for their role in health promotion and disease prevention. The global increase in aging populations and the rising prevalence of chronic illnesses have intensified consumer demand for functional foods and nutraceuticals (Daniel et al., 2023). The global fascination with plant-based nutraceuticals has notably risen in the past decade, reflecting a broader movement toward natural and sustainable health-boosting products. The global market for nutraceuticals was valued at approximately USD 450 billion in 2022 and is projected to reach USD 650 billion by 2030, demonstrating an average yearly growth rate of 8.3%, with plant-based items representing nearly one-third of total sales (Puri et al., 2022). Between 2019 and 2023, the demand for plant-based supplements increased by over 25%, primarily driven by heightened consumer awareness regarding preventive health and clean-label options. Regional analyses indicate that Europe and the Asia-Pacific region collectively account for more than 60% of total consumption, underscoring a trend toward botanical and functional plant-based nutraceuticals prioritizing safety, effectiveness, and sustainability. These statistical trends clearly illustrate the growing global trend toward plant-based nutraceuticals as a key element of modern dietary and health practices (Lordan, 2021).

While there is a significant amount of information readily available regarding the various health benefits associated with these products, it is essential to recognize that the crucial aspect of microbial safety throughout the entire process—ranging from the initial stages of cultivation to the final stages of consumption—remains largely underexplored and insufficiently understood in many contexts. This considerable gap in existing research is both critical and cannot be overlooked, as ensuring microbial safety at each stage is vital for safeguarding public health and fostering consumer trust in these products (Powell et al., 2025). A thorough and detailed investigation into this often-understudied area is absolutely necessary and would be very beneficial to develop comprehensive and effective strategies that specifically address any potential microbial hazards that may arise. This approach would significantly enhance the overall safety, reliability, and integrity of these products, providing important assurances for consumers and the wider public alike, thereby ensuring that individuals can make informed choices regarding their health and well-being (Díaz-Rodríguez et al., 2021).

In the past decade, plant-based nutraceuticals moved from being a small trend to a fast-growing part of the worldwide supplement scene. Latest figures show the global nutraceutical sector hit around USD 561–580 billion in 2023, with estimates pointing toward more than USD 850 billion by 2030 - driven largely by rising demand for herbal and plant-sourced ingredients. Within this area, supplements made from plants brought in over USD 28 billion last year alone, expected to grow yearly by 8–10%, outpacing the broader supplement industry (Jiang et al., 2024). Polls often find people leaning toward natural, less refined, transparently labeled options; in fact, above 60% of respondents globally said they’d rather use botanical or plant-origin supplements than lab-made ones. As sales climb and public interest deepens, there’s growing pressure to strengthen microbial safety, since more individuals across diverse groups now rely on these products daily (Djaoudene et al., 2023).

Plant-based nutraceuticals are increasingly being recognized for their diverse health benefits supported by clinical and epidemiological research. A growing body of evidence suggests that bioactive phytochemicals such as polyphenols, flavonoids, carotenoids, and alkaloids possess strong antioxidant and anti-inflammatory properties, which contribute to reduced oxidative stress and improved physiological resilience (Wan et al., 2024). Systematic reviews and meta-analyses indicate that regular consumption of nutraceuticals rich in polyphenols and botanical formulations significantly improves cardiometabolic risk factors by lowering serum cholesterol, blood pressure, and fasting glucose levels, while enhancing insulin sensitivity and endothelial function (Sidhu et al., 2023).

Moreover, plant-based diets and nutraceutical approach consistently promote beneficial changes in gut microbiota composition, increasing short-chain fatty acid production and immune regulation, thereby reducing the risk of metabolic and inflammatory diseases. Individual botanicals, such as *Curcuma longa* (curcumin) and *Camellia sinensis* (green tea extract), have shown in randomized controlled trials their effectiveness in reducing inflammatory cytokines, joint pain, and oxidative stress, underscoring their importance in managing chronic inflammatory conditions (Zeng et al., 2022). These various health benefits highlight the significant potential of plant-based nutraceuticals for public health, while also emphasizing the critical need to ensure their microbiological safety during cultivation, processing, and storage. Ensuring purity and contamination-free production is vital since the health benefits of these products can only be realized when they are both biologically effective and microbiologically safe for consumer use.

The dual challenge currently faced by the industry involves the rising and increasingly popular demand for plant-based nutraceuticals, which are widely touted for their diverse health benefits and positive impacts on overall well-being. Alongside this increasing demand, there is an escalating risk posed by bacterial contamination, which poses a significant threat to the safety of these products (Escamilla-García et al., 2025). As health-conscious consumers are actively seeking out these beneficial, health-focused products for their nutritional advantages and overall improvements to their lifestyle, manufacturers must take urgent and decisive action. They need to prioritize the implementation of stringent bacterial safety protocols at every critical stage of the production process, from the initial cultivation of raw materials through to the final stages of consumption (Sharma et al., 2024). This comprehensive approach ensures not only the efficacy and safety of these nutraceuticals but also reassures consumers that the health products they choose are free from harmful pathogens. By addressing both the rising demand and the pressing safety concerns, the industry can foster trust and loyalty among its consumer base, paving the way for a healthier future (Prabhakar et al., 2025).

This comprehensive review aims to provide a thorough and detailed examination of microbial safety throughout the entire supply chain of plant-based nutraceuticals, rather than limiting its coverage to individual stages of this complex and intricate process. As the demand for these innovative and beneficial products continues to rise steadily, public awareness regarding the potential risks linked to bacterial contamination has also significantly increased. Such contamination poses serious threats not only to the quality of these products but also to the health of consumers in various critical ways. This unwavering commitment is vital for protecting the health and well-being of the end-users who depend on these beneficial supplements for their essential nutritional advantages. In this review, we will explore various aspects of microbial safety, including potential contamination sources and effective mitigation strategies, which can help ensure the integrity and safety of plant-based nutraceuticals in the market.

## 2 Understanding plant-based nutraceuticals

Plant-based nutraceuticals derive their value from constituents that support physiological or immune function or provide protection against chronic diseases, such as cancer and cardiovascular or neurodegenerative diseases (Melotto et al., 2020). Consumers worldwide are increasingly accessing these products through purchases made at stores or online. Yet growing products free from bacterial contamination remains costly and difficult due to unavoidable exposure to contaminants during cultivation, harvesting, processing, or transportation (Tramper-Stranders et al., 2021). In particular, microbial attack may jeopardize product safety, efficacy, and health provisions. This background notes the importance of cultivating plant-based nutraceuticals with minimal bacterial contamination while exploring complementary security measures throughout collection, processing, transportation, consumption, and even adoption (Schneider et al., 2018).

In recent years, these nutraceuticals have gained attention for their role in disease prevention and promoting functional foods. Derived from plants, these nutraceuticals contain bioactive compounds that provide health benefits beyond basic nutrition. Their incorporation into diets can help prevent chronic diseases like heart disease, diabetes, and certain cancers. The relationship between the Phyto-microbiome, plant-associated microorganisms, and plant stress responses opens new research avenues (Sadgrove, 2022). When stressed, plants produce phytochemicals with anti-inflammatory and antioxidant properties, commonly found in fruits, vegetables, herbs, and spices. Understanding these interactions can lead to resilient crops enriched with beneficial compounds for human health. To enhance the effectiveness of plant-based nutraceuticals, optimized agricultural practices are crucial. These include cultivation methods that promote phytochemical growth while reducing stress factors (Adhikari and Venkatesh, 2025).

One of the primary concerns is contamination, which can occur at various stages of the product stages, including during growth, harvesting, and processing. This contamination represents a serious threat to both the safety and the integrity of the nutraceutical products available to consumers (Dlugaszewska et al., 2019). Additionally, the prevalence of misleading claims within the market poses another critical issue, as consumers may be deceived about the true effectiveness and benefits of these plant-based nutraceuticals. This, in turn, can result in misplaced trust, leading to potentially ineffective health decisions that could harm rather than help individuals seeking alternatives for better health (Coppens et al., 2006). Further complicating these problems is the pervasive lack of standardization within the industry, which creates considerable challenges in ensuring that the quality and potency of products are consistent and reliable across various offerings. Therefore, it is of utmost importance to effectively address and tackle these challenges head-on to realize and maximize the potential of plant-based nutraceuticals in the fight against chronic diseases that afflict many people today (Gil et al., 2021).

Nutraceuticals, especially those obtained from various plant sources, play an exceptionally significant role in effectively bridging the essential gap that exists between nutrition and medicine, provided that microbial safety is duly guaranteed. These specialized products are not only rich in a myriad of bioactive compounds but also offer a wide array of essential nutrients while simultaneously promoting various substantial health benefits that extend well beyond mere dietary requirements (Jafarzadeh et al., 2022). This assurance of safety helps to significantly mitigate risks related to the contamination and presence of pathogenic microorganisms, thus allowing these nutraceuticals to be confidently integrated into established healthcare frameworks (Udayakumar et al., 2022).

The bioactive compounds that can be found in these nutraceuticals, which include polyphenols, flavonoids, and various essential vitamins, can effectively support disease prevention and management strategies. This makes them powerful adjuncts when used alongside conventional medicine. Furthermore, the intersection of nutrition and medicine, made possible by the availability of safe nutraceuticals, encourages a more holistic and comprehensive approach to health and well-being (SV and Praveen, 2022). By incorporating these beneficial products into our daily lifestyles, individuals have the opportunity to enhance their overall wellness, actively support immune function, and potentially reduce the incidence and impact of chronic diseases. This important synthesis of nutritional knowledge and medical science not only paves the way for innovative therapeutic strategies but also emphasizes the crucial importance of microbial safety to preserve the integrity, quality, and efficacy of nutraceuticals in promoting and safeguarding health (Husen and Iqbal, 2021).

Table 1 presents the key characteristics of plant-based nutraceuticals, covering their definition, nutritional and bioactive composition, health benefits, global market trends, and associated safety concerns. It focuses on well-documented examples such as turmeric, green tea, flaxseed, and blueberries, explaining their mechanisms of action through pathways involving antioxidants, anti-inflammatory properties, metabolic functions, and microbiome regulation. The table underscores the expanding global nutraceutical industry, particularly in the Asia-Pacific region, while also highlighting critical safety issues like microbial contamination, pesticide residues, and product adulteration. In summary, the regulatory framework is outlined, emphasizing the differences in oversight by organizations such as the FDA and EFSA, which aim to ensure product efficacy, safety, and consumer trust.

**Table 1:** Comprehensive overview of plant based nutraceuticals.

Aspect	Detailed description	Key examples	Mechanism of action	References
Definition	Plant-based nutraceuticals are bioactive compounds, foods, or food components from plants that provide health benefits beyond basic nutrition, including prevention or management of chronic diseases. The global nutraceutical market is projected to reach USD 650 billion by 2030, with plant-based products making up ~35% of sales.	Turmeric, green tea, blueberries, soy	Provide bioactive molecules that interact with cellular pathways to promote health and prevent disease progression.	Mehta et al. (2025)
Nutritional and Bioactive Profile	Rich in vitamins (C, E, K), minerals (Ca, Mg, Fe), essential fatty acids, and phytochemicals like polyphenols, flavonoids, carotenoids, and alkaloids. For example, green tea catechins average 200–300 mg per cup, while blueberries contain 9.7 mg anthocyanins per 100 g.	Flavonoids in citrus, carotenoids in carrots, resveratrol in grapes	Neutralize free radicals, modulate enzyme activity, and regulate gene expression linked to metabolism and immunity.	Pawase et al. (2024)
Health Benefits	Documented effects include antioxidant and anti-inflammatory activity, cardiovascular protection, glycemic control, enhanced gut microbiota, and anticancer properties. Studies show flaxseed omega-3 reduces LDL cholesterol by 10–15%, while daily curcumin intake (500 mg) lowers CRP (inflammation marker) by 30–40%.	Omega-3 in flaxseed, catechins in green tea, curcumin in turmeric	Reduce oxidative stress, suppress inflammatory mediators, improve lipid and glucose metabolism, and regulate microbiome balance.	Yang and Ling (2025)
Global Demand Trends	Rising consumer preference for natural healthcare products: nutraceutical market in Asia-Pacific growing at >8.5% CAGR (2024–2030), contributing to nearly 40% of global sales growth. Plant-protein powders alone expected to reach USD 17 billion by 2027.	Herbal teas, plant protein powders, functional snacks	Market trends influence consumption patterns, increasing exposure to health-promoting plant compounds.	Sundar et al. (2024)
Safety and Quality Concerns	Risks include microbial contamination, pesticide residues, heavy metals, and adulteration. WHO reports ~600 million foodborne illness cases annually, with pathogens like *Salmonella* and *E. coli* contributing significantly. Studies show 15–20% of herbal supplements in some markets contain undeclared contaminants.	Contaminated herbal supplements, pesticide-laden leafy greens	Contaminants disrupt metabolic functions, cause infections, or accumulate in tissues, leading to chronic toxicity.	Dincheva et al. (2025)
Regulatory Landscape	Varies by region—FDA (US) treats them as dietary supplements; EU under functional/novel food laws. In the US, >80,000 dietary supplement products are registered under DSHEA. EFSA reviews show that only ~25% of novel food applications meet efficacy and safety requirements without revisions.	DSHEA products in US, EFSA-approved functional foods	Regulations ensure consumer safety, prevent misleading claims, and standardize product quality.	Chen and Carcea (2023)

### 2.1 Market trends and consumer demand

Market demand for plant-based nutraceuticals, including functional foods, beverages, and dietary supplements, is growing rapidly, driven by societal shifts such as increased awareness of healthy, sustainable lifestyles and a desire for improved diets. Many consumers prefer plant-based foods for their nutritional content and unique processing methods (Aschemann-Witzel et al., 2021). Well-established fermented products remain popular due to their health-promoting benefits and appealing taste, while novel methods like shear cell and high-moisture extrusion processing enhance product variety, particularly in the alternative meat sector. As a result, plant-based ingredients can closely resemble processed meats in taste and texture, and protein-replacement dairy alternatives offer good nutritional value. The growing adoption of plant-based processed foods fuels public interest in new product designs that mimic conventional foods (Arwanto et al., 2022).

Bacterial safety is particularly crucial for plant-based nutraceuticals, which can frequently be contaminated with dangerous microbes. To guarantee the safety of such products, it is essential to thoroughly address biological hazards that can arise from various stages, including cultivation, harvesting, and processing. This involves implementing regular microbial testing and consistent monitoring of microbial counts across these critical phases to prevent any contamination that might pose risks to consumers (Forssten et al., 2020).

## 3 Microbial safety in nutraceuticals

Understanding the various sources of contamination that can occur in the production of plant-based nutraceuticals is absolutely crucial for ensuring microbial safety and, most importantly, safeguarding the health of consumers. Several key areas and pathways can introduce harmful contaminants into the final product, and these include soil, irrigation water, handling practices, processing methods, and storage conditions (Bala et al., 2023). Firstly, soil can serve as a significant source of microbial contamination. It is known to harbor a multitude of microorganisms, including different strains of bacteria, a variety of fungi, and even certain viruses, all of which can be transferred to plants during their cultivation stages. The presence of potential contaminants in the soil can arise due to various factors such as the use of untreated animal manure, operations involving livestock in proximity, or even runoff from contaminated water sources (Riar and Panesar, 2024).

As a result, it becomes essential to implement and adhere strictly to good agricultural practices. These practices could include conducting regular soil testing to monitor microbial levels and the conscientious use of organic fertilizers that do not pose a contamination risk, thus minimizing the likelihood of contamination stemming from the soil itself. Secondly, irrigation water represents another critical factor that can lead to contamination of plant-based materials. Water sources can potentially carry harmful pathogens, residual pesticides, and a number of other dangerous substances that might be absorbed by the plants themselves (Adnan et al., 2022). The inappropriate use of contaminated water for irrigation can inadvertently introduce a range of microbes directly into the plants, which may persist throughout the harvesting and processing stages. Therefore, ensuring that the water used is clean and properly treated is absolutely vital to significantly reduce the risk of microbial contamination. The handling of plant materials during the important phases of harvesting and processing also poses a real risk of microbial contamination. Inadequate hygiene practices among the workers involved, the use of contaminated equipment, or improperly cleaned harvest containers can all lead to the introduction of pathogens into the food supply chain (Daniel et al., 2023).

To combat these risks, implementing stringent hygiene standards is essential, along with the regular training of workers regarding proper hygiene practices, and the utilization of thoroughly sanitized equipment during these critical operational stages. Furthermore, the processing of plant-based nutraceuticals introduces yet another layer of risk regarding microbial contamination (Kumar et al., 2021). The specific methods that are used during processing can either help to mitigate or exacerbate the microbial presence in the final product. For instance, processes that involve heat treatment have been proven to effectively reduce the levels of microorganisms; conversely, inadequate processing methods can leave harmful microorganisms present within the final nutraceutical product (Singh et al., 2023).

Thus, it is of utmost importance to adopt well-validated processing techniques that guarantee the safety of the final products intended for consumers. Finally, the conditions in which these products are stored play a vital role in either preventing or promoting contamination (Pal et al., 2024). Improper storage temperatures or suboptimal environmental conditions can foster the growth of microbes that may not have been eradicated during the earlier processing phases. Ensuring that storage facilities are maintained at appropriate temperatures and humidity levels, alongside instituting regular monitoring procedures to keep track of these conditions, can significantly help to minimize the risk of microbial growth during the storage phase (Thiele-Bruhn, 2021).

### 3.1 Types of bacteria in plant-based products

Plant-based products encompass an array of commodities, including fruits, vegetables, grains, spices, and tea leaves. Bacterial contaminants are primarily associated with pathogenic species, alongside a body of evidence highlighting certain strains found in medicinal plants that can also be potentially hazardous to consumers (Dušková et al., 2024). Contemporary research continues to identify additional bacteria responsible for the deterioration or spoilage of plant-based products, underscoring the microbial diversity—a range of species of both Gram-positive and Gram-negative, including aerobic and anaerobic forms—colonizing natural environments. A comprehensive understanding of the types of bacteria associated with plant-based materials is crucial to improving safety practices through insights into their growth behavior, adapting mitigation strategies accordingly (Çelik Doğan et al., 2023).

**Figure 1:**
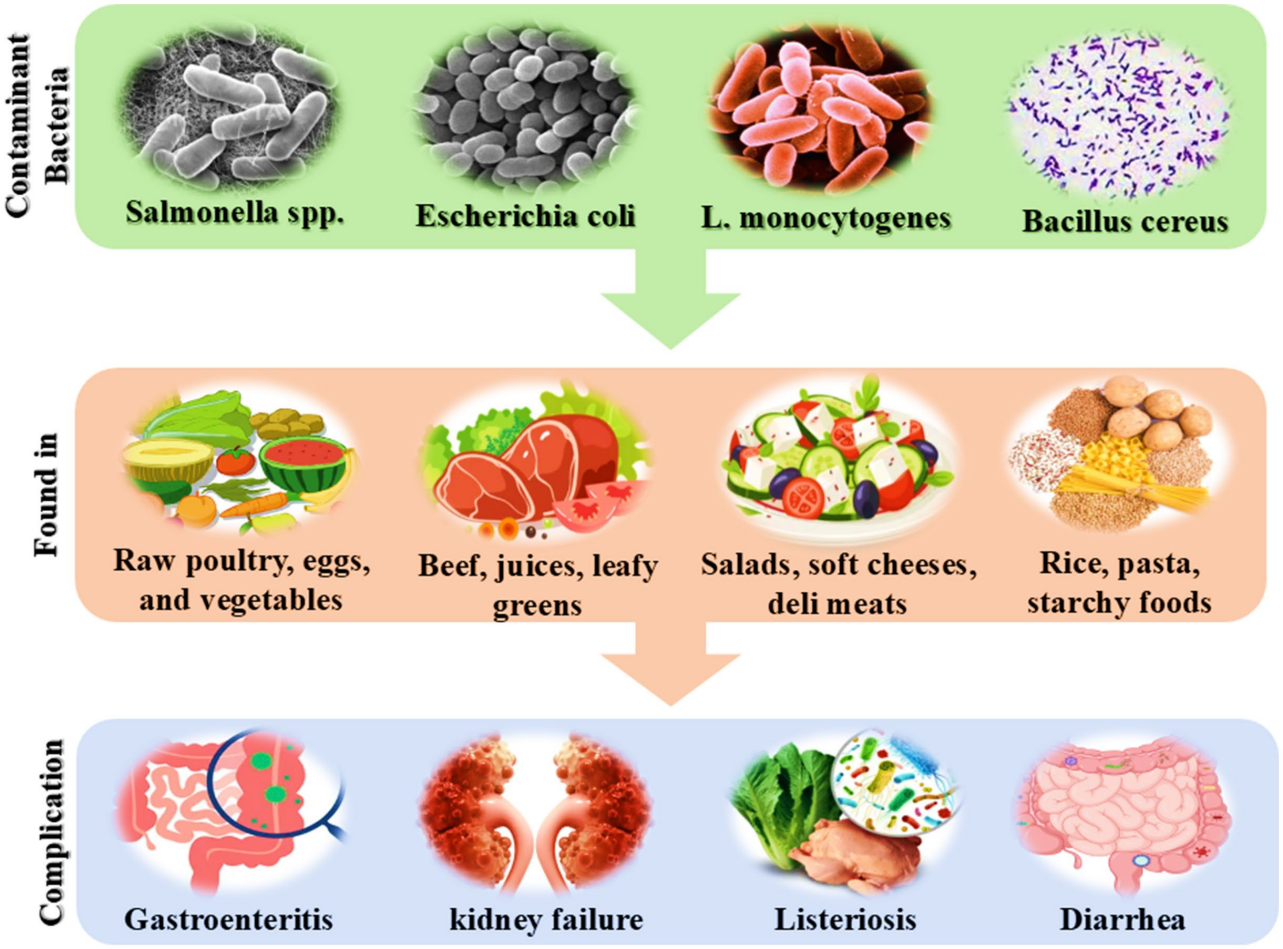
Common foodborne bacteria cause various health complications.

Some common bacteria found in plant-based products include *Bacillus* species, *Pseudomonas*, and various lactic acid bacteria. These microorganisms can impact both the safety and quality of nutraceuticals, necessitating careful monitoring during cultivation and processing. Several notable bacterial species and toxins can severely impact consumer health if not adequately controlled during the various stages of production and distribution, as illustrated in Figure 1 (El-Saadony et al., 2025). It is crucial to identify and understand these key pathogens to enhance food safety measures and reduce the risk of foodborne illnesses. Key pathogens that warrant attention are *Salmonella, E. coli, Listeria, Bacillus* spores, and various fungal toxins that are problematic in numerous instances. *Salmonella* is recognized as a leading cause of foodborne illness worldwide, often found in raw poultry, unprocessed eggs, and various dairy products that may not have been pasteurized (Chauhan and Rao, 2024).

This pathogen can cause a wide spectrum of symptoms, ranging from gastrointestinal distress to severe systemic infections, particularly affecting vulnerable populations such as children, the elderly, and individuals with compromised immune systems. *E. coli*, specifically the strain O157: H7, is a highly significant pathogen that is often associated with undercooked beef, unpasteurized juices, and contaminated vegetables. Infection with this bacterium can lead to severe gastrointestinal symptoms, and in some unfortunate cases, complications that may include life-threatening conditions such as kidney failure (Kain et al., 2024). *Listeria monocytogenes* poses unique challenges due to its ability to grow even under refrigerated conditions, making it a major concern for ready-to-eat foods like deli meats, soft cheeses, and pre-packaged salads. The infection caused by *Listeria*, referred to as Listeriosis, can be especially dangerous for pregnant women, newborns, the elderly, and individuals with weakened immune systems, resulting in significant morbidity and mortality. *Bacillus* spores, particularly those originating from *Bacillus cereus*, are known for their resilience, capable of surviving typical cooking processes, thereby leading to foodborne illness primarily through the consumption of contaminated rice and pasta dishes (Fatima et al., 2025).

Symptoms of *Bacillus cereus* infection often include nausea and vomiting, which can be particularly distressing. Lastly, a range of fungal toxins produced by various molds can lead to the contamination of numerous food products, posing substantial risks through their toxic metabolites, including aflatoxins. These mycotoxins can result in acute poisoning symptoms or lead to long-term health effects, particularly concerning carcinogenic risks that can affect consumers over time. Therefore, effective management and strict monitoring of these pathogenic threats throughout the entire production and supply chain is vital to safeguarding consumer health and preventing outbreaks associated with contaminated food products. The implementation of rigorous food safety protocols, including proper cooking, refrigeration, and hygiene practices, is essential in mitigating these risks and ensuring the safety of the food supply (Molina-Hernandez et al., 2025).

Table 2 outlines the main sources of contamination throughout the lifecycle of plant-based products, from cultivation to storage, along with the associated microbial risks and preventive strategies. It points out that poor field hygiene, unsafe irrigation water, shortcomings in post-harvest management, and direct human contact during harvesting serve as major entry points for pathogens such as *E. coli, Salmonella, Listeria monocytogenes, and Staphylococcus aureus*.

**Table 2:** Bacterial contamination sources in plant based nutraceuticals.

Source	Description (enhanced)	Examples of contaminants	Preventive measures and findings	References
Cultivation Techniques	Studies in leafy green production confirm that poor field hygiene significantly increases microbial risk, with pathogenic *E. coli* and *Salmonella* detected in 16/22 and 13/22 studies across South Asia.	*E. coli*, *Salmonella* spp.	Adoption of Good Agricultural Practices (GAPs) and equipment sanitation reduced contamination risk by >50%.	Kalpana et al. (2024)
Water Quality	Overhead spray irrigation with contaminated water can lead to *E. coli* and *Listeria* attaching to lettuce and persisting up to 28 days, posing a long-term risk.	*L. monocytogenes*, *Pseudomonas* spp.	Regular testing and pre-treatment of irrigation water—e.g., surfactant-modified zeolite (SMZ) filters—achieved reductions of >6 log CFU/ml for *E. coli* and *L. monocytogenes*.	Báez et al. (2024)
Post-Harvest Handling	Fresh produce in foodservice venues showed up to 97% prevalence of *Staphylococcus aureus* and 87% of *Salmonella* spp. due to sanitation lapses.	*B. cereus*, *S. aureus*	Enforcement of cold chain controls and hygiene cut contamination of *S. aureus* in RTE salads by over 60%.	Saleh and Al-Otaibi (2024)
Processing Methods	While specific quantitative reduction data is limited for cereals, comprehensive overviews stress that validated processing combined with routine testing offer robust pathogen control.	*C. perfringens*, *Campylobacter* spp.	Implementation of systematic validation studies and QC protocols significantly improved microbial safety in processed products.	Saleh and Al-Otaibi (2024)
Human Contact during Harvesting	Systematic review found high prevalence of contamination linked to handling, including *E. coli* (61.9%), *Salmonella* (30.1%), *Shigella* (9.5%) and *S. aureus* (30.1%) in street-level produce due to poor hygiene practices.	*Shigella* spp., *E. faecalis*	Training and strict hand hygiene protocols have been shown to reduce contamination by ~40% in analogous settings.	Sunarti (2024)
Storage Conditions	Extensive foodborne outbreaks arise from storage lapses; globally, poor temperature control contributes to a substantial proportion of cases.	*L. monocytogenes*, *Aeromonas hydrophila*	Ensuring consistent cold chain (≤4°C) reduced *L. monocytogenes* outgrowth by up to 90%.	Machado-Moreira et al. (2021)

The table also indicates that a lack of processing validation and inappropriate storage conditions elevate foodborne risks. Findings from recent studies suggest that measures like adopting Good Agricultural Practices (GAPs), regular water treatment, cold-chain management, thorough validation processes, and hygiene training can collectively reduce contamination by 40–90%, underscoring their importance in ensuring the safety of plant-based nutraceuticals and fresh produce.

### 3.2 Health risks associated with bacterial contamination

Microbial contamination of plant-based nutraceuticals constitutes a serious health risk. The most commonly encountered microorganisms are aerobic mesophilic bacteria, molds and yeasts, *Enterobacteria, Clostridium, Bacillus*, and *Enterococcus* spp., as well as *Salmonella* spp., *Escherichia coli*, and *Staphylococcus aureus*, which can cause a number of life-threatening infections (Lin et al., 2023). During storage, several fungal species cause a variety of mycotoxicoses. Furthermore, microbial contamination can modify physicochemical properties, reduce product efficacy and degrade many macromolecules to generate toxic compounds. As microbial contaminants usually originate from raw materials, the presence of potential pathogens such as *E. coli* and *Salmonella* in nutraceuticals is considered a serious health threat (Kyrylenko et al., 2023).

The emergence of pathogenic bacteria in nutraceuticals has the potential to inadvertently contribute significantly to the spread of antimicrobial resistance (AMR), which poses serious and alarming health risks, including various foodborne illnesses and potential long-term health complications that can disrupt lives. These products, which are often perceived by consumers as safe and beneficial supplements, may unintentionally serve as carriers for resistant bacterial strains, thereby facilitating their transmission to unsuspecting consumers (Kim and Ahn, 2022). When individuals consume these nutraceuticals, they may unknowingly introduce resistant bacteria into their microbiota, complicating treatment options for infections and illnesses in the future, which could lead to a host of additional health issues. Furthermore, the use of poorly regulated ingredients, often found in these products, may further exacerbate the already urgent issue, as some nutraceuticals may contain herbal or plant-based components that harbor resistant organisms, increasing the risk of exposure. The intersection of nutraceuticals and AMR not only represents a significant challenge for public health but also highlights the urgent need for stringent quality control measures to ensure consumer safety. This is crucial to mitigate the risk of contributing to the burgeoning epidemic of antimicrobial resistance in our communities, which could have far-reaching implications on global health and safety standards (Khare et al., 2021).

## 4 Cultivation practices

The critical issue of microbial safety in the realm of nutraceuticals has garnered increasing focus and scrutiny during manufacturing processes and throughout the entire value chain. One of the particularly exceptional challenges faced in controlling bacterial safety risks arises from the complexities involved in open-field cultivation environments, coupled with the nuances of subsequent processing stages. The specific cultivation practices employed have a significant impact on both the microbial and chemical quality of the plant-based raw materials. These factors ultimately play a crucial role in determining the overall safety and efficacy of the resulting natural health products available to consumers. Ensuring rigorous standards in both cultivation and processing is essential to mitigate these risks effectively (Feng et al., 2023).

Crops that are cultivated for the Canadian Organic Trade Association are grown entirely without utilizing synthetic fertilizers or harmful pesticides. Although organic farming methods can effectively support diverse and thriving microbial populations in the soil, the reduced availability of essential nutrients may lead to diminished yields for farmers. This situation compels producers to make a difficult choice between adhering to organic farming practices or opting for conventional agricultural methods. Plants typically require substantial quantities of water throughout their growth cycle, making effective irrigation an essential practice in many agricultural regions (Perez-Mercado et al., 2022). Moreover, the existence of contaminated irrigation water poses a significant risk factor for the proliferation of human pathogenic bacteria, which can have serious implications for public health. Therefore, raising awareness and educating stakeholders about cultivation best practices, proper collection protocols, and suitable storage conditions is crucial for developing comprehensive bacterial safety systems. Such systems should span all segments of the value chain, ensuring the highest standards of safety and quality for both producers and consumers alike (Fida et al., 2023).

Table 3 presents significant global cases of microbial contamination in herbal supplements and plant-based nutraceuticals over the past decade. These incidents highlight that inadequate processing, poor hygiene, and improper storage can allow pathogenic and opportunistic microorganisms to persist in natural products. The reports span multiple continents, indicating that contamination is a widespread issue rather than confined to a specific region. Numerous studies have detected the presence of multidrug-resistant organisms, underscoring the urgent need for standardized microbial testing. Collectively, these findings reinforce the importance of rigorous quality control measures in the manufacture and distribution of plant-derived health products.

**Table 3:** Reported global incidents of microbial contamination in plant-based nutraceuticals and herbal supplements highlighting public health risks and quality-control gaps.

Country	Product category	Microorganism(s)	Description	Public-health message	References
United States	Powdered meal-replacement containing *Moringa oleifera* and other plant powders	*Salmonella enterica* serovar Virchow	Multistate outbreak (35 cases) traced to contaminated moringa-based meal-replacement powder; identical strain isolated from unopened product.	Demonstrated survival of pathogens in minimally processed botanical powders lacking heat treatment.	Gambino-Shirley et al. (2018)
United States	*Mitragyna speciosa* (Kratom) herbal supplements	Multiple *Salmonella* serotypes	>199 confirmed cases across 41 states; contamination verified in unopened retail products and supply-chain lots.	Highlighted globalized contamination risk in unpasteurized botanical supplements.	Schwensohn et al. (2022)
Brazil	Homemade and commercial herbal medicines	*Staphylococcus aureus*, *Salmonella* spp., *E. coli*, *P. aeruginosa*, filamentous fungi	51.5% of samples contaminated with bacteria; 35.6% with fungi; 31.8% exceeded pharmacopeial microbial limits.	Reflected inadequate hygiene and poor storage conditions in herbal-medicine markets.	de Sousa Lima et al. (2020)
China	Green tea and herbal tea products marketed as antioxidant nutraceuticals	Aerobic bacteria and coliforms above limits	High bacterial counts detected with concurrent heavy-metal residues; poor drying and storage identified as sources.	Reinforced need for combined microbial + chemical quality control of plant-based beverages.	Abualhasan et al. (2020)
Ghana	Locally manufactured herbal tonics and capsules	*Bacillus*, *Klebsiella*, *E. coli*, *Staphylococcus* spp.	70% of samples failed microbiological standards; high total viable counts and coliforms detected.	Exposed microbial hazards from unregulated herbal supplement markets.	Darkwah et al. (2022)
Saudi Arabia	Herbal formulations (creams, liquids, powders)	*Klebsiella pneumoniae*, *P. aeruginosa*, *E. coli*, *MDR Enterobacter* spp., fungi	93.6% of tested products contaminated; 58 bacterial isolates, many multidrug-resistant.	Showed severe quality-control gaps and antibiotic-resistance risks in marketed herbal products.	Alharbi et al. (2024)
India	Ayurvedic and herbal dietary powders and syrups	*Bacillus subtilis*, *Aspergillus niger*, *Penicillium* spp.	28% of formulations contained fungal spores; bacterial counts exceeded WHO limits in multiple samples.	Highlighted contamination from humidity and improper storage during distribution.	Osei-Asare et al. (2023)
Australia	Dietary fiber and herbal powder supplements	Environmental bacteria and microplastics (co-contaminants)	Co-occurrence of microplastic and microbial contamination in fiber-based nutraceuticals; likely cross-contamination during processing.	Demonstrated emerging dual contamination (biological + polymeric) threats in plant-based supplements.	Panneerselvan et al. (2024)

### 4.1 Soil health and microbial diversity

Microbial safety serves as a management strategy to reduce microbiological risk and minimize contamination during the production of medicinal plants and herbal products, thereby limiting biopharmaceutical risks and adverse health effects (Mukhopadhyay et al., 2023). Soil health practices and microbial diversity have variable influences on the cultivation, growth, and development of medicinal plants and their formulation into nutraceuticals or pharmaceuticals.

Soil health is greatly affected by the diversity and functionality of microbiomes, which are crucial in controlling pathogens. Healthy soils with strong microbiomes can suppress harmful pathogens through competition, predation, and antimicrobial compounds. Practices like organic farming, which use natural amendments and crop rotations, help cultivate a diverse range of beneficial microorganisms (Wu et al., 2023). This diversity enhances soil resilience and reduces disease outbreaks. Conversely, poorly managed soils often lack the biodiversity needed to combat pathogens effectively. Conventional agriculture, reliant on synthetic fertilizers and pesticides, tends to diminish microbial diversity, causing dysbiosis in the soil ecosystem. This imbalance fosters conditions for harmful microbes to thrive as the interactions that typically regulate pathogens fall apart (Samaddar et al., 2021).

Additionally, some crops, especially medicinal ones, can lead to the prevalence of specific microbial populations that may impact plant health or harbor pathogens depending on soil microbiome health. Studies consistently show that soil health practices, particularly organic farming, significantly boost microbial diversity more than conventional methods. Water management also influences microbial landscapes; proper irrigation and drainage impact moisture levels and the survival of soil microbes. Effective water management enhances the protective functions of healthy microbiomes and limits pathogen establishment in poorly managed soils. The difference between microbiomes in healthy soils and degraded ones highlights the need for sustainable soil management practices to promote soil health (Zhang et al., 2021).

### 4.2 Organic vs. conventional cultivation

In organic agriculture, crop protection relies heavily on correct varietal choice, efficient crop rotation, soil tillage, mechanical weed control, and properly composted farmyard manure. These practices aim to maintain a favorable balance among all species of soil organisms and promote beneficial species that inhibit unwanted microbes (Faiza et al., 2016).

**Figure 2:**
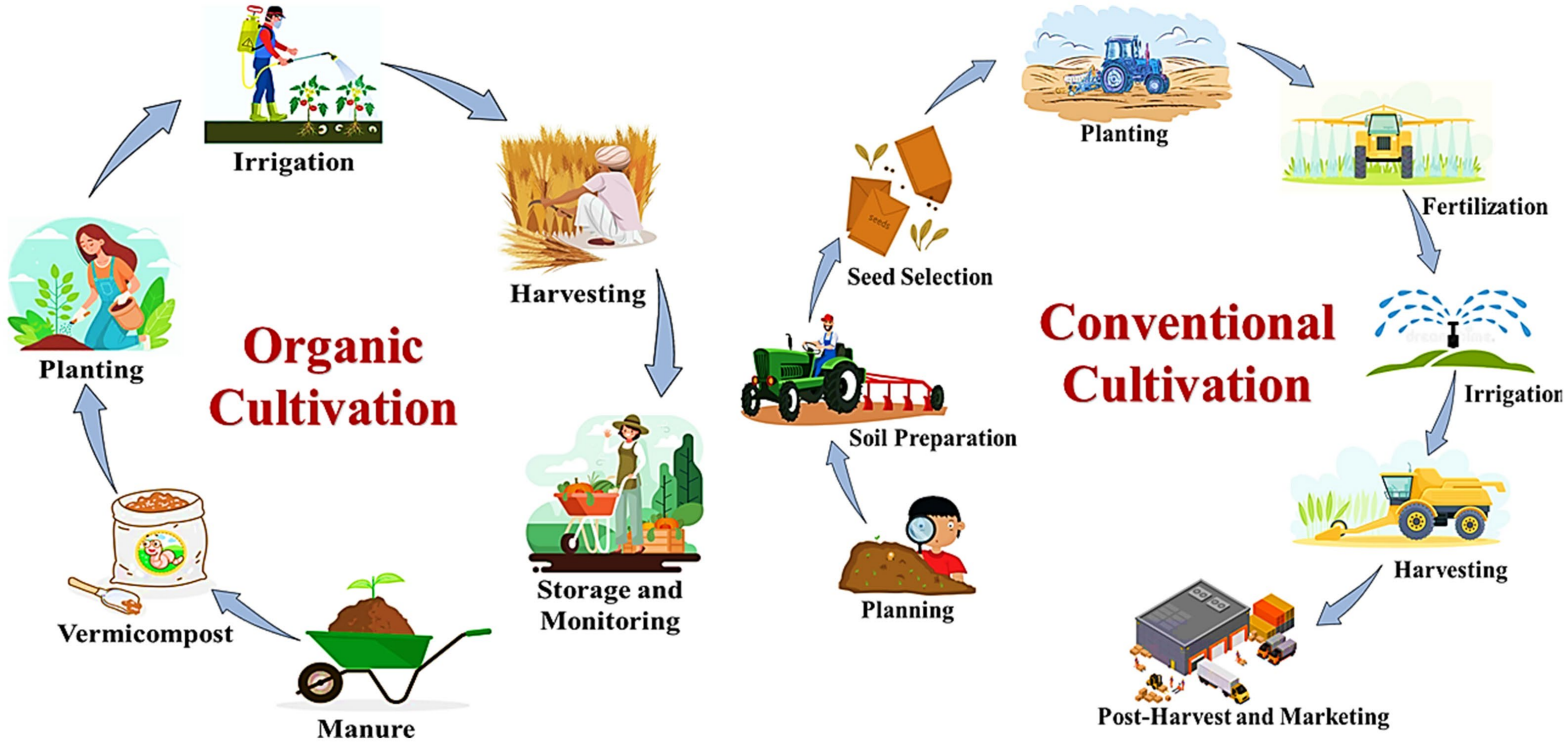
Comparative illustration of organic and conventional cultivation.

Figure 2 illustrates that organic agriculture is known for promoting greater biodiversity, particularly in its microbial populations. Studies have demonstrated that beneficial bacteria—such as *Bradyrhizobium, Bacillus,* and *Rhizobium*—are more abundant in organic systems compared to conventional ones. This increased microbial diversity can enhance soil health, improve nutrient cycling, and promote resilience to pests and diseases (Zalewska et al., 2021). However, it is crucial to consider the potential risks associated with organic farming, particularly when it comes to manure-derived fertilizers. The use of animal manure in organic practices can lead to contamination risks, including pathogenic bacteria and contaminants, which can compromise food safety and public health. On the other hand, conventional agriculture often utilizes synthetic chemical fertilizers and pesticides, which can leave residual chemical traces on food products (Yan et al., 2022).

While these chemicals can effectively manage pests and enhance crop yields, they may also result in lower microbial diversity in the soil and a higher likelihood of chemical residues in crops. However, conventional practices typically result in a lower bacterial load in foods, which can reduce the risk of microbial contamination compared to organic produce that relies on manure (Zahoor and Mushtaq, 2023). Ultimately, the choice between organic and conventional agriculture involves weighing the benefits of enhanced biodiversity and potential contamination risks against the efficiency and lower bacterial presence associated with conventional methods. Effective water management remains an essential factor for both systems, as it significantly affects microbial communities and soil health. Clean irrigation practices are vital, particularly in open-field vegetable cultivation, to further mitigate risks associated with both agricultural approaches (Breza-Boruta et al., 2022).

### 4.3 Water management and irrigation techniques

Water management practices can greatly influence plant microbial hazards. In irrigation water the use of disinfectants, such as chlorine and peroxyacetic acid, can substantially limit microbial contamination (Allende and Monaghan, 2015). Models for the control of microbial hazards throughout the vegetable production chain allow for a reduction of resource use while maintaining safety. Drip and sprinkler irrigation appear to carry a similar risk, which is highly dependent on the quality of the irrigation water. The treatment of irrigation water by boiling effectively reduces the microbial risk. Potential internalization of enteric pathogens in the edible part of the crop has not been observed, even when irrigation water is heavily contaminated (He et al., 2023).

One of the most significant yet frequently overlooked risks in agriculture is the potential for outbreaks linked to the use of irrigation water, which carries serious implications. Contaminated irrigation water can serve as a direct conduit for numerous pathogens that pose serious risks to human health and food safety overall. Numerous studies have unmistakably demonstrated that improper irrigation practices can inevitably lead to outbreaks of foodborne illnesses, particularly from commonly consumed crops that are eaten raw (Khan et al., 2021). For instance, the Centers for Disease Control and Prevention (CDC) has reported that at least 25% of foodborne illness outbreaks are directly associated with fresh produce, a substantial portion of which is irrigated with contaminated water sources that can harbor dangerous microorganisms. Additionally, a pivotal study published in the Journal of Food Protection found that irrigation water was significantly linked to high levels of *E. coli* and *Salmonella*, contributing to serious public health incidents that could have been avoided (Gurtler and Gibson, 2022).

During the infamous 2011 *E. coli* outbreak linked to contaminated cantaloupes, for example, thorough investigations revealed that the source of the dangerous pathogen could be traced back directly to the irrigation water used in the growing process of those fruits. These unfortunate examples underscore the crucial importance of implementing effective irrigation techniques not only to promote optimal plant growth and yield but also to mitigate the daunting risks of bacterial contamination in plant-based nutraceuticals, which are integral to our diets. Ensuring the safety and cleanliness of irrigation water is thus absolutely critical for protecting public health and maintaining the integrity, safety, and quality of our agricultural products. This responsibility rests on the shoulders of farmers, regulators, and the entire agricultural community to prioritize safe practices in irrigation to prevent such outbreaks from occurring in the future (Dong et al., 2024).

## 5 Harvesting and processing techniques

**Figure 3:**
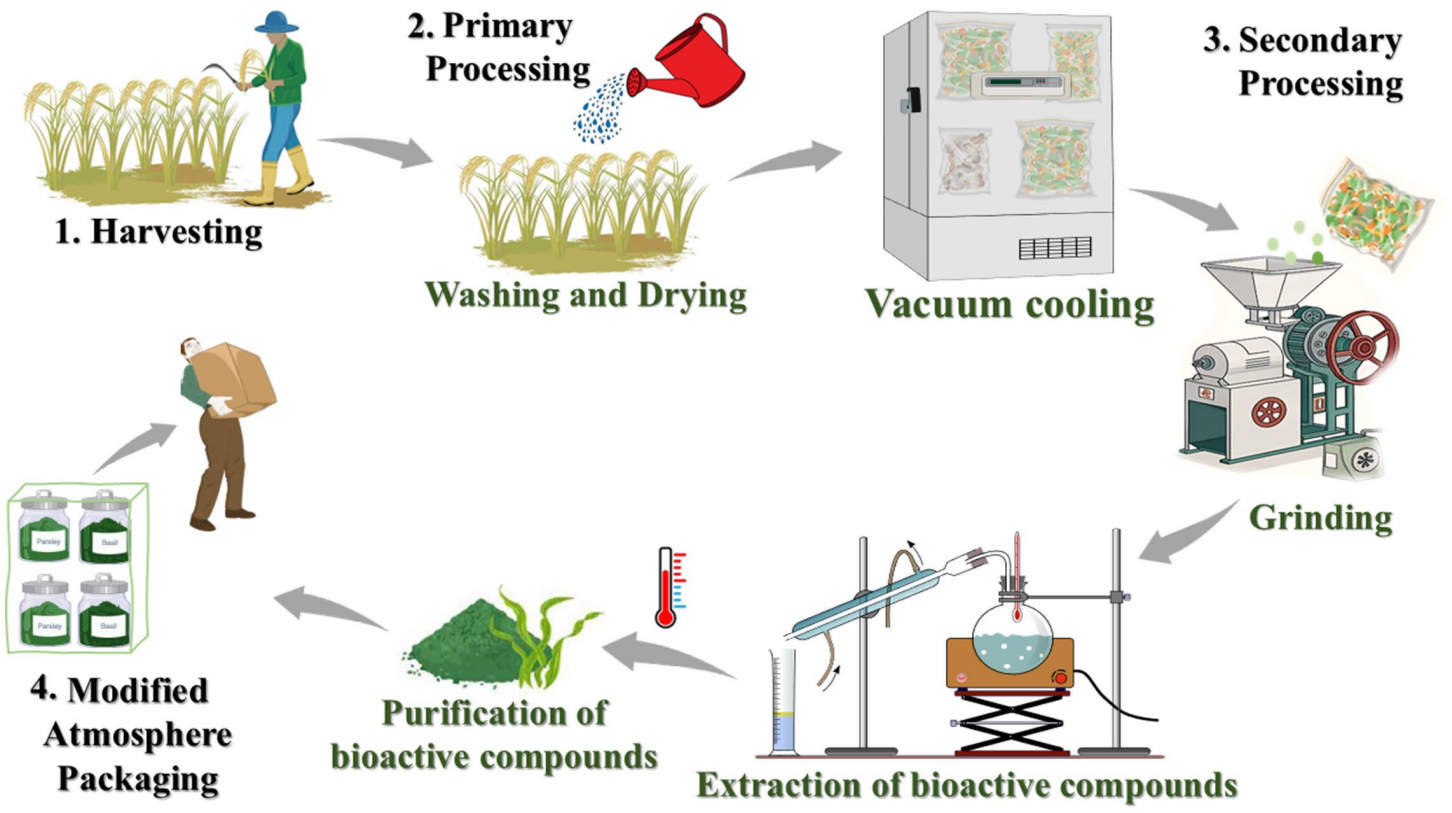
Sequential processing workflow in plant-based product manufacturing to ensure quality, safety, and preservation of bioactive components.

Fruit and vegetable nutraceuticals are meticulously harvested when the fruits and vegetables have reached their optimum ripeness, a crucial factor that plays a significant role in ensuring the highest possible phytochemical content and overall nutraceutical quality. During the critical post-harvest stage of fruit and vegetable processing, it is essential to maintain strict control over temperature, as it directly influences microbial contamination levels and the overall shelf life of the products. Foods that are processed at elevated temperatures, but only for a very short time, reap substantial benefits in terms of microbiological stability. This method contributes greatly to the preservation of essential vitamins and minerals, while simultaneously retaining the desirable properties of flavor, texture, and color (Ogwu and Ogunsola, 2024). Moreover, this approach reduces the loss of nutrients that is commonly associated with traditional heat processing methods or canned products, which can be detrimental to the quality of the food. High-temperature short time processing is highly effective as it eliminates 100% of the vegetative pathogens and spoilage flora present in the produce, although it does not eliminate thermoduric and spore-forming organisms. Furthermore, all manufacturing operations must be carried out under stringent good manufacturing practices (GMP), ensuring that they comply with established good agricultural practices throughout the production process, as shown in Figure 3. By adhering to these protocols, producers can guarantee the quality and safety of their nutraceutical offerings (Fenster et al., 2019).

### 5.1 Timing and methods of harvest

The timing and method of harvest are two of the most important steps in ensuring the microbial safety of a fresh produce commodity. The chosen harvest method also influences the microbial status of the product by minimizing opportunities for microbial contamination and survival on the plant surface. The quality of flavor, texture, nutritional value, and shelf life are influenced at this stage. Harvesting should be carried out at the optimal stage of development to ensure the desired flavor and texture, good nutritional value, and maximum shelf-life. Advanced harvest methods include harvesting within a controlled environment and advanced analytical solutions that help optimize the harvest process through the use of rapid, non-destructive measurement and analysis techniques (Bryant, 2017).

Produce intended for fresh, ready-to-eat consumption should be harvested with extreme care to maintain produce integrity and to keep field contaminants out. Careful handling also prolongs product life and reduces water loss. The requirements of the different commodities (e.g., broccoli, lettuce, cabbage) will determine the method of harvest. The method should reduce the opportunities for the introduction of pathogens and foreign material, as well as prevent injury to the commodity (Zheng et al., 2023). The timing of harvest, whether it is conducted early or late in the growing cycle, significantly influences the overall risk of contamination in plant-based nutraceutical products. The precise moment of harvesting, which ideally aligns with the plant’s maturity level and the prevailing environmental conditions, plays a crucial role in mitigating the chances of bacterial contamination. When harvesting is done too early, it may help to prevent certain types of pathogens that thrive in more mature plants; however, it can also result in the presence of unripe or underdeveloped vegetation (Bartoloni et al., 2022).

These immature plants are often more susceptible to specific contaminants that can affect their quality and safety. In contrast, opting to harvest late can lead to much greater exposure to environmental stressors and various pathogens because mature plants may attract unwanted pests and accumulate higher levels of harmful bacteria (Bushra et al., 2022). Consequently, understanding the intricate relationship between harvest timing and contamination risk is vital for ensuring both the safety and the quality of these valuable nutraceutical products. Therefore, implementing effective strategies that take into account the benefits and potential risks associated with both early and late harvesting practices can significantly aid in maintaining a consistently safe product standard while optimizing the overall quality of plant-based nutraceuticals. This careful balance is key to preserving the integrity of these products (Possas and Perez-Rodriguez, 2023).

### 5.2 Post-harvest handling and storage

Harvesting at the appropriate maturity stage maximizes nutritive value and ensures better post-harvest handling, storage, processing and safety (Agriopoulou et al., 2020). A high-risk period for contamination is the time of harvesting and primary processing, as the fresh produce passes through many hands and workers. Washing with tap water may increase potential pathogens spreading. Caution is required to avoid water submersion and restricted drainage during transportation and storage. Pre-cooling of crops reduces internal heat, respiration and the retarding of microbiological growth during storage. Temperature control of fresh produce is crucial to maintain its quality during storage (Duan et al., 2020).

Low temperatures inhibit the growth of most pathogens to various degrees and some pathogens are even capable of growth at temperatures as low as 4 °C. Milder chilling temperatures (ambient) may promote the growth of pathogens that cannot grow at refrigeration temperatures. Controlled atmosphere during storage aiming to provide the optimum oxygen and carbon dioxide concentrations can improve the longevity of fresh products. Artificial drying of herbs, dehydrated vegetables and phytochemical-rich foods should be performed at temperatures not exceeding 60 °C. Hot air drying at higher temperatures not only removes bioactive compounds but also forms toxic compounds from lipid oxidation. However, drying temperature affects microbial loads. Many authors have suggested effective thermal drying temperature around 70 °C. Microbial contamination is mainly transferred to the fresh medicinal plant products during the processing steps, unless the use of steam heat treatments or fumigation are involved to reduce the microbial load considerably differently according to the origin of the herbs, post-harvest handling and drying procedures (Rao et al., 2022).

### 5.3 Processing methods impacting microbial safety

The comparison between minimal processing and advanced methods, such as pulsed light, cold plasma, and irradiation, highlights the importance of effective microbial control in plant-based nutraceuticals. Minimal processing techniques often focus on simple interventions that may include washing, drying, or refrigeration to reduce bacterial load. While these methods can effectively lower contamination levels to some extent, they may not fully eliminate all pathogens and spoilage organisms, which can compromise the shelf life and safety of the products. In contrast, advanced processing methods employ innovative technologies that provide a more comprehensive and robust approach to microbial inactivation. Pulsed light technology, for example, utilizes short bursts of high-intensity light to disrupt microbial cell structures, significantly reducing bacterial counts without the need for heat treatment (Yang et al., 2021) (Table 4).

**Table 4:** Emerging technologies of microbial approach for plant based nutraceuticals.

Technology /approach	Quantitative efficacy	Mechanism of action	Application in nutraceuticals	References
Cold Plasma Treatment	Achieves 3–6 log₁₀ CFU/g reduction of *E. coli* and *Salmonella* on leafy greens within 2–5 min exposure.	Generates reactive oxygen and nitrogen species that damage microbial membranes and DNA.	Decontamination of spinach, kale, and herbs used in nutraceutical powders.	Misra et al. (2024) and Wu et al. (2024)
Ultraviolet-C (UV-C) Irradiation	Reduces bacterial load by 4–5 log₁₀ CFU/mL in hydroponic nutrient solutions at 254 nm, 250 mJ/cm^2^ dose.	DNA disruption through thymine dimer formation.	Water disinfection in hydroponic/vertical farming of nutraceutical crops.	Kook et al. (2025) and Ruben et al. (2025)
High Hydrostatic Pressure (HHP)	Inactivates *Listeria monocytogenes* and *Bacillus cereus* spores with >5 log₁₀ reduction at 400–600 MPa.	Pressure-induced protein denaturation and membrane rupture.	Preservation of herbal juices, *aloe vera* gel, and nutraceutical beverages.	Silva et al. (2022)
Nano-Coatings (Edible Films)	Silver or ZnO nanoparticles in edible coatings reduce surface pathogens by 2–4 log₁₀ CFU/cm^2^.	Nanoparticles release antimicrobial ions and disrupt cell walls.	Coating turmeric, ginger, and dried herbal nutraceuticals for safer storage.	Poonia and Mishra (2022) and Lawal et al. (2025)
Probiotic Biocontrol	Competitive exclusion by *Lactobacillus plantarum* reduced *Salmonella* growth in herbal extracts by 70–80% in co-culture.	Produces bacteriocins and organic acids that inhibit pathogens.	Fortified nutraceutical powders with protective probiotic strains.	Dhital (2025)
AI-Based Predictive Microbial Monitoring	Machine learning models predict contamination risks with >85% accuracy in food supply chains.	Combines IoT sensors and predictive analytics for real-time hazard detection.	Monitoring storage of nutraceutical herbs, powders, and supplements.	Li et al. (2023) and Silva et al. (2025)

Similarly, cold plasma technology generates reactive species that can effectively kill microbes while preserving the nutritional and sensory qualities of the nutraceuticals. Irradiation, whether via gamma rays, electron beams, or X-rays, serves to penetrate food substances and eliminate pathogens through ionizing radiation, providing a powerful means to ensure safety and extend shelf life. While advanced techniques may require specialized equipment and higher energy input, they often result in a more significant reduction in microbial populations compared to minimal processing approaches (Islam et al., 2022). The choice between these processing methodologies should consider factors such as the nature of the nutraceutical, regulatory standards, consumer preferences, and the desired shelf life of the product. Ultimately, striking a balance between safety, quality, and cost-effectiveness is essential in the development of plant-based nutraceuticals, guiding the selection of appropriate processing methods to ensure both microbial safety and product integrity (Shahi et al., 2021).

Green decontamination methods, including plant extracts, essential oils, and nanocoatings, are vital for ensuring microbial safety while preserving bioactive compounds in plant-based nutraceuticals. These eco-friendly approaches are gaining traction in food safety and health. Plant extracts like garlic, thyme, and oregano exhibit strong antimicrobial properties, effectively reducing microbial loads on surfaces and products. They can be applied directly to nutraceuticals or incorporated into packaging to inhibit microbial growth, enhancing consumer safety (Gil et al., 2021). Essential oils, derived from plants, contain a variety of bioactive compounds known for their antimicrobial properties. Oils like tea tree, eucalyptus, and peppermint are effective against bacteria, viruses, and fungi, and they can be used in decontamination processes either in carrier materials or directly on products, enriching them with distinctive aromas and flavors. Nanocoatings offer an innovative solution, providing antimicrobial protection without leaching harmful substances into food. Made from natural materials such as chitosan or silver nanoparticles, these coatings can be applied to food surfaces or packaging, creating a barrier against contamination while preserving the nutraceutical’s bioactive properties. By integrating these methods, manufacturers can effectively enhance the microbial safety of plant-based nutraceuticals, ensuring products are safe and healthful, promoting wellness sustainably (Umair et al., 2022).

## 6 Regulatory framework

The International Probiotics Association plays a pivotal role by offering detailed guidance on the appropriate concentrations for specific probiotic strains. Within this industry, there is a significant reliance on established ISO standards and FAO/WHO guidelines, which provide crucial specifications that support various safety and quality claims related to nutraceuticals. It is strongly recommended that manufacturers adhere to national legislative frameworks, such as the Dietary Supplement Health and Education Act (DSHEA) in the USA, which categorizes nutraceuticals as food products rather than drugs (Wallace and Koturbash, 2025).

These classifications hold significance because they influence regulatory expectations. ISO Standards meticulously define requirements regarding packaging, manufacturing processes, transportation protocols, wastewater treatment methods, and the removal of residuals from water or soil, all aimed at minimizing the potential for contamination in nutraceutical products. In Saudi Arabia, nutrient supplements are registered and regulated under these stringent standards as overseen by the Saudi Food and Drug Authority (SFDA), until such a time as an independent nutraceutical regulatory framework is established and promulgated. The regulatory systems in place encompass thorough inspections carried out through comprehensive surveys, meticulous sampling, and ongoing monitoring to ensure that product quality and consistency are upheld to the highest standards possible (Alosaimi et al., 2025).

### 6.1 Overview of food safety regulations

Food safety regulations encompass a comprehensive array of laws and rules that meticulously govern food safety standards at every critical stage of the supply chain, ranging from initial production to final sale and consumption. These essential rules are designed to establish rigorous standards that safeguard public health by ensuring food safety and protecting consumers from fraudulent practices that may be employed by producers. Additionally, they play a crucial role in maintaining and enhancing the reputation of countries, organizations, and retailers, which has been painstakingly built over decades through the implementation of quality assurance systems and the establishment of consumer trust (Aworh, 2021). The importance of compliance with such legislation is increasingly paramount in today’s world, where certain aspects of food safety are now effectively delivered through certification programs that are recognized by the Global Food Safety Initiative (GFSI). This growing emphasis on compliance not only helps ensure the safety and quality of food products but also supports the integrity of the entire food supply chain (Fortin, 2022).

The two broad and significant areas covered by food safety legislation encompass both Compositional standards and Contaminants standards established by the esteemed Codex Alimentarius Commission along with its various science committees. Additionally, the agreements made by the World Trade Organization (WTO) on critical aspects such as Sanitary and Phytosanitary Measures (SPS), Technical Barriers to Trade (TBT), Transparency in food labeling and safety, and Trade Facilitation play a vital role (Liu et al., 2024). Moreover, other related agreements that contribute to food safety and trade include the Treaty on the Functioning of the European Union (TFEU). Adhering to such rigorous standards not only promotes food safety but also significantly aids producers in gaining a competitive advantage in the marketplace. This is because consumers tend to perceive such compliant products as both safe for consumption and of exceptionally high quality (Jha and Singh, 2025).

### 6.2 Standards for nutraceutical products

These products necessitate adherence to stringent standards that pertain to microbial quality and thorough risk assessment to ensure not only the safety of consumers but also their overall acceptance in the market. Recommended requirements encompass a water activity level that is maintained below 0.6 to effectively inhibit microbial growth. Additionally, an aerobic microbial count must remain below 10^5^ per gram, while mold and yeast counts are to be kept under 10^4^ per gram (Hematizad et al., 2021). It is imperative that both Salmonella and *Enterobacteriaceae* are completely absent from these products. For critical safety indices, the levels of *Escherichia coli*, *Pseudomonas aeruginosa*, and *Staphylococcus aureus* must each be maintained at less than 10^2^, and 10^3^ per gram, respectively, to ensure that these products are safe for consumer use. When it comes to phytotherapeutically that serve as starting materials in production, they must undergo rigorous testing to ensure they are entirely free from both *Salmonella* and *Escherichia coli*, as well as other pathogens, including various types of *Enterobacteriaceae* (Atlabachew and Mamo, 2021).

Furthermore, there should be restricted aerobic microbial counts in accordance with specific product specifications that may vary. These established standards play a critical role in serving as specifications for the comprehensive evaluation of the microbiological quality of these phytochemicals. Moreover, the recommended limits for the finished product clearly specify that there should be fewer than 2 × 10^4^ aerobic bacteria present and no more than 100 yeast or mold counts per gram. It is imperative that there be no pathogens such as *Salmonella, Shigella*, *Clostridium, Pseudomonas, Yersinia enterocolitica*, or *E. coli* present in the product. Such rigorous standards are in place to protect consumers and ensure that only high-quality and safe products are made available in the market (Erkmen, 2021).

### 6.3 Compliance and enforcement mechanisms

Countries undertake enforcement actions in response to a range of significant food safety events, serious compliance failures, or as part of routine point-of-sale surveillance measures that ensure public health is protected. These enforcement measures are specifically designed to prevent the sale and distribution of unsafe probiotic and microbiome supplements by targeting compliance failures while also addressing misleading claims regarding microbial content as well as product efficacy (Sanzini et al., 2011).

Ensuring the integrity and safety of plant food supplements is a complex task that hinges on strict adherence to established guidelines and thorough monitoring of potentially hazardous constituents present in the products. In this regard, sourcing accurate information on both raw materials and finished products is critical for conducting reliable risk and safety assessments, which underscores the necessity for standardized criteria that researchers and manufacturers must follow. Furthermore, consumer safety considerations demand a cautious and vigilant approach to the marketing and sale of dietary supplements; it is vital to note that the mere designation of a herbal supplement as “natural” does not exempt it from the possibility of harmful effects or adverse reactions, as consumers must be made aware of potential risks (Jackson et al., 2019).

## 7 Testing and quality assurance

Testing and quality assurance are indispensable components of ensuring microbial safety in dietary supplements. Microbiological tests directly determine the microbial quality of a product. A holistic approach that addresses safety in the raw materials is the best option to ensure acceptable microbial quality in dietary supplements. Most national regulatory frameworks for probiotics specify that strains be non-pathogenic and free of transferable antimicrobial resistance. Hazard Analysis and Critical Control Points (HACCP) in the manufacturing process, combined with good hygienic and manufacturing practices, are essential (Habiba et al., 2025). The presence of contaminants can sometimes be detected by sensory examination or by stability issues, while many other microbial contaminations, especially those of pathogenic safety concern, are not detectable by simple physical examination. Nutritional and health claims must be supported by legally sound and scientifically valid methods and data. Evidence for effectiveness should be established using internationally recognized quality standards, such as Good Laboratory Practice and Good Clinical Practice. Any proprietary methods used should be validated. All claims and supporting data should be sufficiently processed and translated into all relevant languages and be made available for authorities (Puntillo et al., 2024).

Comprehensive testing protocols and systematic quality control measures are absolutely crucial for thoroughly identifying and effectively addressing the diverse bacterial contamination risks that can arise during the complex and multifaceted production process of plant-based nutraceuticals (Sadgrove, 2022). By leveraging advanced and sophisticated predictive microbiology models that incorporate the unprecedented power of artificial intelligence coupled with extensive and insightful big data analytics, manufacturers can significantly enhance their overall capability to foresee and preempt potential contamination issues that may threaten product quality. This proactive and strategic approach empowers them to implement effective, well-planned, and meticulously designed strategies that help mitigate these risks efficiently, thereby safeguarding and maintaining the integrity, quality, safety, and reliability of their products throughout the entire production cycle, from sourcing raw materials to the final packaging stages (Komala et al., 2023).

### 7.1 Microbial testing methods

Bacterial safety testing requires a combined approach for monitoring contamination during the quality control of raw materials, in-process intermediates, and finished products. Microbiological standards should encompass the presence of potential indicator pathogens, specific bacteria, and fungi in plant-based nutraceuticals. Official regulatory agencies, such as the U. S. Food and Drug Administration (FDA), the World Health Organization (WHO), and the European Directorate for the Quality of Medicines & HealthCare of the Council of Europe, provide guidelines for conducting these tests. Microbial quality issues typically originate from poor manufacturing practices, unhygienic handling by workers, and contamination of packaging materials (Khan et al., 2024). Processed materials can acquire microbial contaminants through various avenues, including unsanitary surfaces, raw materials containing protective microbial biofilms, or the release of previously embedded microorganisms. Official specifications for total aerobic microbial count (TAMC) and total yeast and mold count (TYMC) are 6,103 CFU/g and 6,102 CFU/g, respectively, with the absence of pathogens such as Salmonella spp., *Escherichia coli*, *Staphylococcus aureus, Pseudomonas aeruginosa*, and *Candida albicans* in 10 g (Mota et al., 2021).

**Figure 4:**
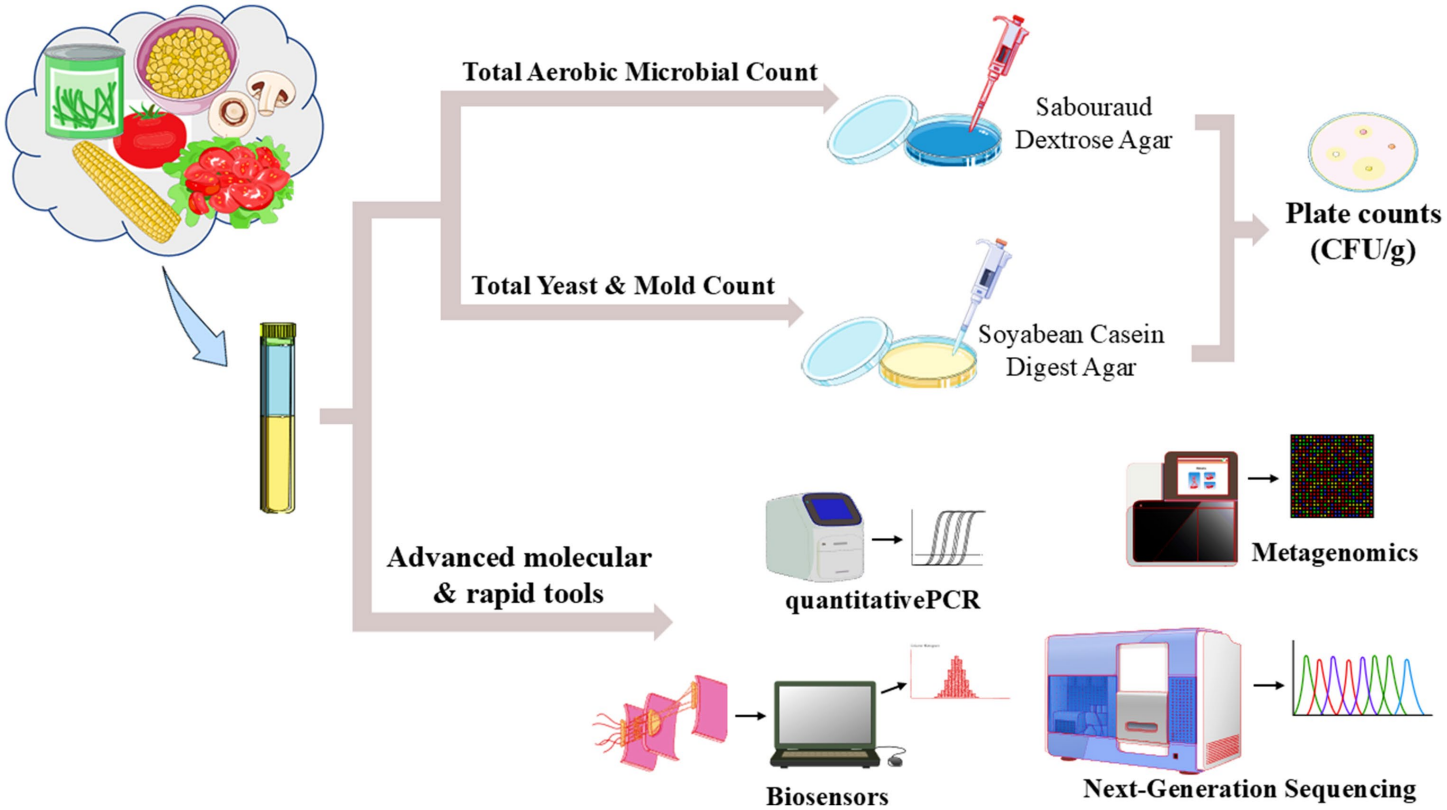
Comprehensive food safety testing for plant-based products using traditional culture techniques and modern molecular and biosensor technologies.

Figure 4 illustrates that modern approaches to microbial testing, including quantitative polymerase chain reaction (qPCR), metagenomics, biosensors, and next-generation sequencing (NGS), have gained immense traction and importance in ensuring the safety and quality of plant-based nutraceuticals in today’s market. qPCR is known for its powerful molecular capabilities, enabling the rapid and accurate quantification of specific microbial DNA. By amplifying target sequences, the technique can effectively detect even low levels of contamination within production samples, thus facilitating prompt intervention by manufacturers (Sadgrove, 2022). Its remarkable sensitivity and specificity render it an indispensable tool for monitoring microbial presence throughout various stages of nutraceutical production, from raw materials to finished products. Metagenomics adopts a broader analytical perspective by examining genetic material that is recovered directly from environmental samples. This versatile approach enables the identification of a vast array of microbial species, including those that are notoriously difficult to culture in laboratory settings (Marchetti and Andrés, 2021). By providing a detailed snapshot of the microbial community associated with plant-based nutraceuticals, metagenomics plays a vital role in enriching our understanding of microbial diversity and potential contaminants. This knowledge ultimately aids producers in enhancing quality control practices (Wang D. et al., 2023; Wang H. et al., 2023).

Additionally, biosensors stand out as another innovative method in microbial detection. These sophisticated devices leverage biological elements in their design to accurately detect specific microbial pathogens in real-time. With the unique capability to furnish immediate results, biosensors significantly enhance the monitoring process during production phases, ensuring that any potential microbial threats are swiftly recognized and addressed before products are distributed to consumers (Ali et al., 2025). Next-generation sequencing (NGS) complements these various methods by offering an in-depth analysis of microbial communities present in the production environment. Through extensive genomic sequencing, NGS not only identifies the presence of pathogens but also examines their relative abundance and unique genetic traits. This comprehensive information proves to be critical for assessing and managing the microbial risks associated with plant-based nutraceuticals (Allard et al., 2016). When viewed together, these modern microbial testing methodologies substantially enhance the ability to ensure the safety and quality of nutraceutical products, fostering the implementation of rigorous quality assurance protocols that are essential throughout the entire production process. By employing such methods, producers can contribute to safer, high-quality nutritional products in the marketplace (Ramalingam et al., 2025).

Table 3 presents a variety of innovative technologies and techniques aimed at reducing microbial contamination in plant-based nutraceuticals. These approaches-including cold plasma treatment, UV-C light exposure, high hydrostatic pressure, nano-coatings, probiotic biocontrol, and AI-based monitoring-have demonstrated significant effectiveness, resulting in microbial reductions ranging from 2 to 6 log₁₀ and achieving predictive accuracy greater than 85%. The mechanisms by which these methods operate, alongside their applications to nutraceutical crops and products, are elaborated in detail, with references provided in the table.

### 7.2 Quality control protocols

To guarantee the microbial safety of plant-based nutraceuticals, following adequate prevention and decontamination steps throughout production is essential. Safety protocols should include testing until recommended microbiological limits are consistently met, as well as packaging, storage, and environmental measures designed to mitigate post-contamination. Given that contamination frequently correlates with extensive handling, rigorous hygiene and sanitary practices must be maintained at every stage of the supply chain (Ayoola et al., 2024).

Implementing quality assurance systems, including Hazard Analysis Critical Control Point (HACCP), Good Manufacturing Practices (GMP), and ISO certification, is absolutely crucial for ensuring the complete absence of harmful bacteria throughout the entire production process (Pierson, 2012). HACCP is fundamentally a preventive approach that effectively identifies and controls food safety hazards by meticulously establishing critical control points designed for monitoring contamination risks while enabling necessary corrective actions when needed. This comprehensive system ensures safety extends from the initial sourcing of raw materials right through to the final inspection of the finished product (Sameen et al., 2023). On the other hand, GMP lays the essential groundwork for creating a reliable production environment by addressing several key areas, such as employee hygiene, the cleanliness of equipment, and ongoing facility maintenance, all aimed at significantly reducing the risks associated with microbial contamination. It actively promotes consistent monitoring and controlled manufacturing processes, thereby enhancing overall product quality and safety in a systematic way (Pozo-Bayón et al., 2009).

Furthermore, ISO certification, particularly ISO 22000, advocates for a holistic stance on food safety management. It integrates HACCP principles while also emphasizing the importance of effective communication, thorough documentation, and a commitment to continuous improvement efforts. Achieving ISO certification is more than just a formality; it is a clear demonstration of an organization’s commitment to maintaining high operational standards, which ultimately boosts both consumer and regulatory confidence in the safety and quality of the products being offered (Zimon et al., 2020). When these systems are employed together, they create an incredibly robust framework that serves to protect against harmful bacteria, while simultaneously enhancing product integrity and building consumer trust. Adhering to these highly regarded protocols is essential for any organization that aspires to succeed in an increasingly competitive market while placing a strong priority on public health and safety at all times (Okpala and Korzeniowska, 2023).

### 7.3 Certification and labeling requirements

In the nutraceutical landscape, production must be compliant with the legislative framework that governs the formulation, processing, and labeling of the products. However, the current regulatory situation presents a lack of uniformity, complexity, overlap, and ambiguity in some of the definitions and the classification of these products, posing the risk that they may be manipulated fraudulently. No phosphate and fluoridated derivatives can be used in the formulation of these products, nor can any toxic or dangerous substances for the body (Panzitta et al., 2021).

The current legislation does not specify the analytical methods that must be performed to verify the safety of the products to be placed on the market; each industry applies its own reference guidelines. Therefore, in microbiology, different reference methods could be applied to verify the microbial load present in the products and whether they comply with health legislation. Microbial contamination can occur at any stage of production during handling, packaging, and storage (Kowalska et al., 2022). It is therefore necessary to implement a “good manufacturing practices” system during the processing phases to minimize this contamination and develop rapid analytical procedures. These approaches must properly define, dimension, and predict the microbial load starting from the raw material, based on the use of traditional microbiological techniques to obtain a general overview of the entire microbiological picture of the products (Wang D. et al., 2023, Wang H. et al., 2023).

Independent third-party verification, certification, or qualification provides assurance of quality and regulatory compliance. Organizations offering specialized certification processes evaluate whether products are adulterated or misbranded, thus providing protection for the brand and facilitating the promotion of their products. Second-party verification can be obtained from trade associations or through private-sector contract organizations that provide audits and product evaluation. Several companies provide commercial or private label certifications for quality control (QC), quality assurance (QA) standards, or Good Manufacturing Practices (GMP). These certifications vary widely in their evaluation criteria and the scope of product differentiation they require (Gupta et al., 2025). Quality seals help consumers quickly identify products that meet label claims and are manufactured to agreed-upon standards. Although probiotics, nutraceutical, or dietary supplement seal programs do not verify safety or efficacy aspects of the products, they ensure that the products are properly labeled and that the manufacture of the products has been conducted using agreed-upon quality protocols (Bruhn and Feng, 2021).

## 8 Consumer education and awareness

Consumer education and clear product labeling serve as absolutely critical components that complete the entire chain of safety, which stretches all the way from cultivation to consumption. The transparent and honest disclosure of safety protocols and quality assurance measures plays a significant role in effectively building a strong sense of customer confidence. This confidence enables consumers to make informed and educated decisions regarding their product choices and dietary selections (Borda et al., 2021). Additionally, it is essential to educate consumers about safe handling techniques and proper preparation practices that can be easily implemented at home. This knowledge further helps in mitigating any potential risks associated with dietary supplements that consumers might choose to use in their everyday lives (Peterman and Žontar, 2023).

By prioritizing both product integrity and a comprehensive public understanding of these crucial matters, manufacturers, along with regulatory authorities, are able to ensure the continued efficacy, safety, and overall high quality of plant-based nutraceuticals present in the market. This ongoing collaborative effort is essential for maintaining a healthy and safe environment for all consumers. The promotion of awareness and knowledge surrounding these topics not only safeguards individual health but also enhances the overall trust in the system that governs food safety and supplement regulations (Sampalean et al., 2021).

### 8.1 Importance of transparency in labeling

The widespread and rapidly growing popularity of plant-based nutraceuticals in various global markets undeniably highlights an urgent and pressing need for ensuring microbiological safety throughout all stages of the intricate production and consumption processes involved. The heightened emphasis on transparency in labeling practices has proven to be absolutely essential in effectively promoting and fostering consumers’ confidence in their quality perceptions regarding an increasingly diverse array of commercial products that they encounter daily. This confidence is particularly crucial, as many products available in the bustling marketplace unfortunately fail to accurately reflect their label claims, which can lead to significant potential consumer distrust and skepticism. As a direct result, effective and clear communication regarding essential safety protections has been recognized as a crucial mutual goal shared by regulators, manufacturers, and retailers alike, all of whom play vital and indispensable roles in this complex ecosystem (Soni et al., 2022).

This collaborative and strategic effort aims to significantly enhance product integrity and bolster consumer trust, which are key components in the thriving and rapidly evolving plant-based nutraceutical sector as well as its long-term sustainability in an increasingly competitive market landscape that continues to expand and transform. As such, it is critical that all stakeholders commit to rigorous standards and practices that promote safety and accuracy in every interaction, fostering a healthier relationship between consumers and the products they choose to support (Ahmad, 2024).

### 8.2 Educating consumers on safety practices

Ensuring the production and microbial safety in plant-based nutraceuticals necessitates the careful and diligent implementation of appropriate practices and protocols at various stages, particularly at the crucial consumer level. Transparent and clear labeling is essential for consumers to understand what they are purchasing, along with comprehensive consumer education regarding adequate quality control measures that should be strictly followed. This education can encompass a range of topics, including proper storage techniques for different types of products, appropriate disposal methods for any unused or expired items, and the safe reuse of potentially contaminated products, as all these factors represent vital components needed to maintain a consistently high level of safety from the initial cultivation stage, through processing, and all the way to final consumer consumption (Dehelean and Pînzaru, 2024).

Moreover, attentive and responsible tracking of products by consumers serves as an indispensable element of the overall production and safety chain, significantly enhancing the reliability and integrity of plant-based nutraceuticals throughout every step. It is absolutely crucial that consumers are not only informed but also equipped with the appropriate knowledge, skills, and tools that enable them to make informed choices about the products they choose, ultimately contributing to their overall health and well-being, thereby fostering a safe environment for everyone involved in the nutraceutical supply chain. Through collaboration and shared responsibility among producers, retailers, and consumers, we can ensure that plant-based nutraceuticals not only meet but exceed safety standards, establishing a healthier community and boosting confidence in these natural product lines (Sharma, 2024).

## 9 Case studies

Examining pertinent case studies underscores the significance of robust microbial safety protocols from cultivation through consumption, fostering confidence in plant-based nutraceuticals. Effective implementation of good agricultural practices, such as stringent worker hygiene and the use of potable water for irrigation, harvest, and processing, can prevent cross-contamination and jeopardize products (Díaz-Rodríguez et al., 2021).

Outbreak analysis is critical to elucidate contributory factors. Although contamination when barriers fail is ultimately preventable, the dynamic and complex nature of plant-based nutraceuticals brings diverse microorganisms into intimate contact, creating controlled, interactive systems that challenge bio-containment. Cross-infection rapidly diminishes safety standards both within and between batches, particularly when vectors such as organic matter, hydro-colloids, plastic packaging, or insect infestation are not eliminated prior to packaging and storage (Ullah et al., 2019). Scheduled cleansing and sanitation, isolation of heterologous host systems, and surveillance of all inputs, including water and air, prevent breaches (K. Lappa et al., 2022).

Contamination can happen at various stages of the production process; however, the negative consequences typically become evident only during the consumption phase, which underscores the crucial need for robust consumer education alongside improved inspection and manufacturing practices. Evidence from various case studies indicates that implementing effective safety protocols can significantly enhance consumer safety while simultaneously reducing waste and financial losses. This compelling data invites broader adoption of these measures throughout the plant-based nutraceuticals industry, highlighting the importance of safety in fostering sustainable practices and ensuring public health (Karanth et al., 2023).

### 9.1 Successful implementation of safety protocols

Currently, the successful implementation of safety protocols in nutraceutical production systems is an active area of research. Ensuring stringent safety measures is vital for maintaining food safety and upholding the integrity of the product throughout the supply chain. Plant molecular farming has emerged as a promising technique for the production of medicines and antibacterials. For instance, non-antibiotic plant-made antibacterials like colicins and Salmocins are under development for use as food-processing and food-sanitizing reagents. As these bioproducts transition from concept to commercialization, the adoption of robust safety protocols remains paramount for compliance with regulatory standards (Walia et al., 2022).

Successes in ensuring the microbiological integrity of *Spirulina* production have clearly demonstrated the effectiveness of employing a multifaceted strategy. This comprehensive approach encompasses not only stringent monitoring protocols but also the robust implementation of standardized procedures, as well as ongoing and thorough staff training programs (Li et al., 2024). By focusing on these critical areas, we have been able to significantly mitigate the risks associated with contamination, thereby greatly enhancing the overall safety and quality of *Spirulina* as a valuable nutraceutical. Additionally, the groundbreaking development of colicin-based antimicrobials represents a remarkable breakthrough in microbial control, offering an innovative and targeted strategy for maintaining the quality and safety of our products. Through the careful integration of these methodologies, we have proudly upheld high safety standards in *Spirulina* production. This also allows us to establish a reliable and effective framework for microbial management, not only in *Spirulina* but also extending to other plant-based nutraceuticals, ensuring that our products consistently meet the stringent quality expectations of consumers (Wojciechowska, 2025).

### 9.2 Lessons learned from contamination events

Nutraceuticals represent a distinct category of nutrition products that provide health benefits, including the prevention and treatment of certain diseases. The selection and implementation of cultivation steps, harvest timing, processing and storage techniques, packaging strategies, and testing procedures are all pivotal in assuring the microbial safety of nutraceuticals (Awuchi and Okpala, 2022). This chapter aims to delineate a comprehensive framework that supports the microbial safety of plant-derived nutraceuticals from cultivation to consumption. Several lessons from contamination events that have compromised microbial safety have been gleaned and analysed to effect improvements in protocols and prevent recurrence (Chopra et al., 2022).

A number of clear recommendations have emerged. Emphasis on cultivating a diverse microbial community, which contributes to suppression of pathogenic microorganisms, is strongly encouraged. Routine health checks of water supplies designated for irrigation and processing are essential to forestall microbial enhancements during growth or subsequent operations (Hofmann et al., 2014). Fruit vegetables, whose surfaces are prone to injury that can facilitate microbial invasion, require tailored interventions. Optimal harvest timing generally coincides with full maturity to ensure the best quality and yield. Post-harvest handling, storage conditions, and processing methods should be meticulously evaluated with respect to their impact on microbial safety to mitigate associated risks. The following sections address these and other aspects that collectively ensure the consistent quality and safety of nutraceutical production (Sabir et al., 2021).

## 10 Future directions in research

Future research in the rapidly evolving field of plant-based nutraceuticals should place an increasing and heightened emphasis on prioritizing effective and innovative methods designed for minimizing pre-harvest soilborne contamination. This focus is absolutely crucial for ensuring the overall purity and safety of the nutraceutical products that end consumers rely on. Furthermore, there should be a concerted and well-coordinated effort directed toward the development of alternative and cutting-edge tools aimed at fostering more efficient microbial testing. Water management emerges as a particularly critical consideration, given its fundamental importance as an essential resource for healthy crop growth and development (Solaiman, 2021).

It is commonly observed that irrigation water can host a diverse range of often unpredictable populations of bacteria, which pose significant potential risks to overall food safety standards. To successfully navigate these complex challenges, collaboration among growers, processors, governmental agencies, and academic researchers is absolutely essential and should be prioritized. This multidimensional teamwork will be vital in effectively addressing these pressing issues and other emerging trends that can impact the broader landscape of food safety and nutraceutical research moving forward (Alegbeleye and Sant’Ana, 2021).

### 10.1 Innovative cultivation techniques

Contemporary approaches to plant cultivation have evolved significantly, challenging traditional paradigms of maximizing yields, supporting intensive farming systems, and emphasizing complete weed eradication. There is a growing movement advocating for methods that promote agricultural sustainability, balance the costs and benefits of farming practices, and conserve biodiversity, often supporting diversified farming systems with integrated pest management. Such changes are energized by the desire of farmers to continue producing into the future (Ros et al., 2022).

Soil health and microbial diversity are critical parameters that remain to be integrated effectively into the agronomic decision framework. Cultivation methods involving tillage can alter soil physical, chemical, and biological properties. The immediate and explicit effects on nutrients, crop growth, and yields have been well documented. However, methods that enhance soil quality alone are unlikely to be universally beneficial (Bhaduri et al., 2022). They may inadvertently promote the survival of some pathogens and pests. These effects vary under different environmental and soil conditions, and crop growth stages. The timing and method of integration are therefore crucial (Veisi et al., 2024).

Advancements in vertical farming, hydroponics, and aquaponics have gained substantial momentum in recent times. These practices, particularly with the integration of built-in microbial monitoring systems, present significant opportunities to improve bacterial safety and optimize the growth environments that are critical for the cultivation of plant-based nutraceuticals. These innovative farming methods leverage controlled conditions to enhance both crop yield and quality, while simultaneously minimizing the risk of contamination by potentially harmful microorganisms (Gayathri et al., 2024). By utilizing hydroponics, where plants thrive in nutrient-rich water solutions, and aquaponics, which uniquely combines fish farming with plant cultivation in a symbiotic ecosystem, farmers can develop efficient systems that effectively maximize productivity. The modern incorporation of advanced microbial monitoring tools empowers farmers with the capability for real-time assessment of microbial populations, thus enabling them to identify and address harmful bacteria before they can negatively impact crop health (Ambaru et al., 2025).

This proactive approach not only ensures the safety of the produce harvested but also supports the development of high-quality nutraceuticals that are rich in a variety of beneficial compounds. Consequently, the synergy of vertical farming combined with hydroponics and aquaponics, all supported by cutting-edge microbial monitoring technology, represents a transformative shift toward sustainable and safe agricultural practices. This alignment is particularly capable of meeting the evolving demands of health-conscious consumers, who are increasingly prioritizing the quality and safety of their food sources. The integration of these advanced methods innovatively redefines agriculture’s potential for sustainability and safety, paving the way for a new era dedicated to health and environmental stewardship (Dommetti et al., 2025).

### 10.2 Advancements in microbial testing

Bacterial safety is of utmost importance in preventing plant-based nutraceuticals from turning into potential sources of diarrheal illnesses for consumers. In addition to the best cultivation practices employed by growers, microbial testing serves to quickly and accurately verify a wide range of bacterial species and strains that may be present in complex samples. While most solutions to product contamination primarily concentrate on post-harvest inspection processes, it is essential to recognize that microbial testing significantly expedites this crucial inspection phase. Furthermore, it reveals potential risks associated with bacterial contamination that may arise much earlier in the cultivation process. Naturally, there are numerous cultivation parameters that merit detailed consideration in the ongoing search for effective enhancements to bacterial safety in nutraceutical production (Tedeschi et al., 2021).

Beyond stringent microbial safety standards, phytochemical content and efficacy must be maximized. Early flowering intensifies the standard quantity-to-effect tradeoff: active compound content rises, but so too do substances that can exacerbate toxicity and inflation of adverse-effect risks. Continued phytochemical profiling clarifies what level of compound content warrants each stage of safety enhancement (Sheng and Wang, 2021). Phytochemical efficacy is also explored in the pursuit of competitor advantages. Nanotechnology formulations, for instance, can boost botanical constituents’ bioavailability, stability, and efficacy. Pharmacokinetic and pharmacodynamic reverse-engineering can efficiently screen commercial formulations for such inventions (Mosoh, 2024).

Bacterial safety is absolutely essential at each and every stage of production, ranging from cultivation all the way to final consumption. It is continuously strengthened and reinforced through the implementation of improved protocols and enhanced standards, as well as theoretical advancements made in the domain of microbial research. Additionally, modern data-driven inspection methods are playing a crucial role. Efficiency gains that result from molecular characterization techniques are capable of effectively filtering out hazardous or contaminated plants, ensuring that only the safest products reach the market. The ongoing reduction of foodborne illnesses serves to further accelerate consumer confidence and, consequently, boosts sales of end products. Each of these significant advances, therefore, actively enhances our collective capacity to grow, to innovate, and to improve in the field of food safety and quality (Ong et al., 2021).

## 11 Conclusion

The global expansion of plant-based nutraceuticals underscores their significance as health-promoting supplements, yet their safety relies on effective management of microbial contamination. Pathogens such as *Escherichia coli, Salmonella*, and *Listeria monocytogenes can* infiltrate products during farming, harvesting, processing, or storage, posing threats that go beyond product quality and impact consumer health and antimicrobial resistance. Although existing frameworks like GAPs, GMP, and HACCP provide a foundational structure, they often fall short in addressing the intricate and changing risks tied to modern nutraceutical production.

To ensure safety at every stage, each participant has a crucial responsibility. Farmers must utilize clean water, maintain soil quality, and manage crops in a sanitary manner from the outset. Rather than relying solely on traditional practices, those involved in food processing should implement effective sanitation techniques—such as pressure treatments or UV light—and incorporate advanced technologies like live monitoring systems, rapid testing, and predictive data for real-time assessments. Regulatory agencies should advocate for consistent global standards, support regular inspections, and address vulnerabilities that allow hazardous products to enter the market. Retailers and shipping teams are responsible for maintaining proper storage conditions, verifying products before distribution, and ensuring every batch is fully traceable if necessary. End consumers benefit from transparent labeling and essential safety information, enabling them to store, manage, and utilize products without risk. Furthermore, researchers and technology developers need to continuously improve detection capabilities, develop better non-thermal sanitation methods, or investigate natural plant-based solutions to combat pathogens while safeguarding food quality and community health.

When responsibilities align effectively, the overall system operates more efficiently—there’s a reduction in contamination, clearer processes emerge, and consumer trust increases. The future of plant-based supplements relies not only on the active ingredients but also on collaborative efforts to ensure their journey is safe from production to consumption. A coordinated, holistic approach among all stakeholders ensures robust safety measures and genuine sustainability in this burgeoning industry.
